# Long-Term Study of the Relationship between Precipitation and Aquatic Vegetation Succession in East Taihu Lake, China

**DOI:** 10.1155/2017/6345138

**Published:** 2017-02-02

**Authors:** Yehui Zhang, Na Yang, Jiawei Xu, Yixing Yin

**Affiliations:** College of Hydrometeorology, Nanjing University of Information Science & Technology, Nanjing, China

## Abstract

Based on long-term rainfall measurements (1956–2012), water level records (1956–2006), and aquatic plants field survey data (1960–2014), the relationship between precipitation and aquatic vegetation succession in east Taihu Lake, China, is studied. Neither abrupt changes nor any trends were found in the annual rainfall series in Taihu Lake during the studied period (1956–2012). However, for seasonal variations, statistically significant decreases are found in spring and autumn, while the rainfall in winter exhibits statistically significant increase. No significant trend was obtained in summer. A “dry” period was detected in our studied period (1963/1964~1978/1979). Total annual rainfall was significantly positively correlated to the number of rain-days (*r* = 0.59) and the water level (*r* = 0.84). Our results indicate that the variations of rainfall and water level may have an impact on the aquatic plants in Taihu Lake. The dry period may be not suitable for the growth of the aquatic plants. All aquatic plants in Taihu Lake were dramatically reduced in the dry period, especially for submerged macrophytes and floating-leaf macrophytes. Our results may be helpful for the aquatic restoration in the future.

## 1. Introduction

The formation, development, and maintenance of wetland ecosystems are closely related to the water resources [1, 2]. The excessive utilization of water resources directly leads to the dryness of wetlands and eventually the degradation of natural wetlands [3, 4]. Therefore, overall planning of water resources is important for the stable and healthy operation of natural/restored wetlands [5]. In order to fully understand and effectively manage the wetland ecosystems, data accumulations (including both physical and biological variables) are required in various scales and under contrasting climatic and biophysical condition [6–8]. However, researches in relationships between long-term observations of water dynamics (e.g., precipitation and/or water level) and plant vegetation responses are relatively rare, especially in humid lands such as wetlands.

As a functional group, aquatic plants are one of the most important components in aquatic ecosystems [9, 10]. Aquatic plants are the main producers of oxygen, the basis of the food chain and a major part of the energy flow in the aquatic ecosystem [11]. The plants form a complex spatial habitat that provides food and sanctuary for organisms living in and around, stabilizes sediments and participates in matter cycling, and regulates wetland hydrological conditions [12]. Many recent researches have pointed out that the response of aquatic plants, especially submerged macrophytes, is associated with the changes in water levels [13–15]. Several models have been developed to predict vegetation responses as a function of annual, seasonal, or monthly rainfall or of rainfall-related indices [16–18]. Unfortunately, most of the current studies related to precipitation are from terrestrial ecosystem. The effect of long-term precipitation on aquatic vegetation has not been fully understood in most cases due to the lack of sufficient data to conduct such long-term assessments.

Since the beginning of the industrial era, anthropogenic greenhouse gas emissions (largely driven by economic and population growth) and the increasing greenhouse gas concentrations in the atmosphere have led to various changes in the Earth's climate at an unprecedented rate [19]. According to the IPCC fifth report [20], global warming is an indisputable fact. The global average surface temperature (including land and ocean) has shown a warming temperature of 0.85°C (0.65 to 1.06°C) over the period 1880 to 2012, and the increase in temperature is likely to continue and even intensify [20]. Climate change may lead to a large variation in interannual and intra-annual rainfall [21], plus extreme weather/climate events (e.g., droughts, floods, and extreme precipitation), which would affect the structure and function of global ecosystems and the sustainable development of many human systems.

Global climate change will directly lead to changes in precipitation and the rainfall imbalance. In general, precipitation will be decreased in the low latitude areas, while it will be increased significantly in the high latitude regions [22]. However, there is a certain degree of uncertainty in global climate model. For instance, Piao et al. [23] analyzed data from 355 precipitation monitoring sites in China since 1960. It is found that there is no significant change in the total rainfall over the long-term period in China. But significant regional precipitation trends are found. The rainfall in drier regions of northeastern China is decreasing in summer and autumn. In contrast, the wetter area of southern China is receiving more rainfall in both summer and winter. Such trends may result in droughts in the northern part of China and flooding in the southern part of China, which will significantly change the coverage and species composition of wetland vegetation and soil physical and chemical characteristics [24].

Taihu Lake is the third-largest freshwater lake in China, occupying a surface area of 2,425 km^2^ and the average depth is about 1.9 m. The lake is located in the core of the Yangtze Delta within the lower reaches of the Yangtze River Basin, which is the most developed areas in China. Due to the rapid socioeconomic development, the aquatic ecosystem of Taihu Lake has degraded. The distribution and community structure of aquatic vegetation have clearly been changed. The main aim of this paper is to investigate the relationship between rainfall patterns and aquatic vegetation-related processes based on the point of view of plant ecology, by using the long-term (1956–2012) rainfall records from the weather station in Taihu Lake, Dongshan (Suzhou, Jiangsu province, China).

## 2. Data and Methods

**Figure 1:**
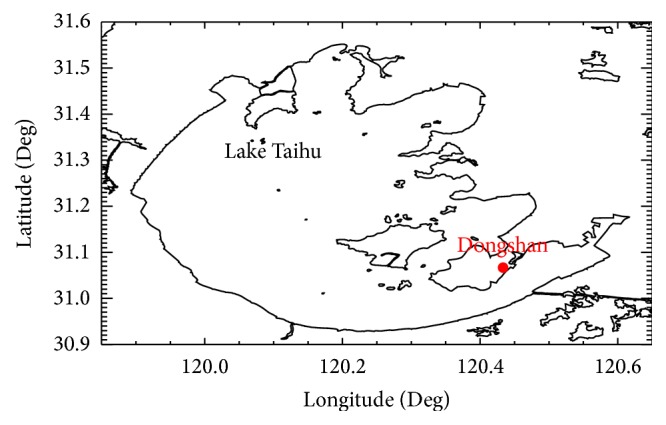
Location of the Dongshan weather station.

The long-term observations used in this paper are from the Dongshan weather station, which is freely available from the National Meteorological Information Center (http://data.cma.cn/). The Dongshan weather station (31°4′N, 120°26′E) is located near east Taihu bay (Figure 1), with the altitude of 17.5 m above sea level. The data ranges from 1956 to 2012. For each measurement, meteorological variables such as pressure, temperature, relative humidity, rainfall, and winds are recorded. Regarding the rainfall record, it measured one-day precipitation depth from 20 LTC (local time) to 20 LTC. Besides the rainfall data, the data of annual water level are also used in this paper, which is from 1956 to 2006. The average annual temperature at Dongshan station is 16.5°C, the average total annual rainfall is 1127.7 mm (1956–2012), and the average annual water level of Taihu Lake is about 3.09 m (1956–2006).

The monthly rainfall values, which are calculated from daily rainfall data, are used to obtain the seasonal and annual rainfall values. Regarding the rain-day, when rainfall volume was ≥1 mm, it was considered to be a rain-day. Annual and monthly rain-day averages were then calculated. Following the method described in Lázaro et al. [25], rainfall was considered “normal” between the first (25th) and the third (75th) quartile, under 25th as “dryer” than normal and over 75th “wetter” than normal (Table 1). The 10th and 90th percentiles were considered thresholds for extreme values. This criterion is commonly followed to characterize “very dry,” “dry,” “normal,” “rainy,” and “very rainy” climatic conditions.

The cumulative sums of deviations are calculated to investigate the possible nonabrupt changes in the long-term rainfall records and differentiate wet and dry periods. It can be calculated as follows:

(1) $$\begin{array}{c} S_{k}=\overset{k}{\underset{i=1}{\sum }}d_{i},\;k=1,2,\ldots ,n. \end{array}$$

A change in the slope of *S*_*k*_ denoted a climatic change in the long-term series. The occurrence of the maximum |*S*_*k*_| represented a change point. The advantage of this method is that it can detect the invisible changes with strong variability in rainfall or runoff series [26, 27].

To investigate the possible existence of abrupt changes and trends, two methods are adopted in this paper. The first one is the sequential version of the Mann–Kendall test proposed by Sneyers [28], which is applied for the annual series. The Mann–Kendall test is a rank-based nonparametric method. It is thought to be suitable for data not with a normal distribution, which is usually used in rainfall series. It is based on the following procedure. Considering a time series of length *n* (*x*_*i*_, where *i* = 1,2,…, *n*), for each *x*_*i*_, the number *r*_*i*_ of *x*_*i*_ > *x*_*j*_ with *j* < *i* is computed. Then, the sum is calculated:

(2) $$\begin{array}{c} s_{k}=\overset{k}{\underset{i=1}{\sum }}r_{i}. \end{array}$$

This sum presents a normal distribution with an average (*E*) and variance (Var):

(3) Esk=kk−14,varsk=kk−12k+572,k=2,3,…,n.

Then, the UF statistic is calculated as follows:

(4) $$\begin{array}{c} {UF}_{k}=\frac{\left[s_{k}-E\left(s_{k}\right)\right]}{\sqrt{var\left(s_{k}\right)}},\;k=1,2,\ldots ,n \end{array}$$

and UF_*k*_ is compared with a standard normal distribution at the required level of significance, which is usually *α* = 0.05 [28, 29]. The null hypothesis is rejected when |UF_*k*_| > 1.96. UF_*k*_ > 0 indicates an increase and UF_*k*_ < 0 indicates a decrease.

By using the retrograde series of *x*_*i*_ and also going through the whole procedure above, the UB_*k*_ statistic is obtained. If curve UF_*k*_ or UB_*k*_ passes through the 5% significance level from inside outwards, it is considered that the trend is significant. In addition, if curves UF_*k*_ and UB_*k*_ clearly cross each other (not overlap) between the critical values at the 5% level, there is an abrupt change, with the intersection point representing the beginning of that change.

The second trend estimate approach was computed for each of the four three-month seasons (December-January-February, DJF, etc.) using data for the period 1956–2012. Trends were computed using the nonparametric median of pairwise slopes method [30], with statistical significance levels based on Spearman rank-order tests.

There has been extensive amounts of aquatic vegetation field survey data collected from east Taihu Lake. In the current study, we collected a series of published field survey papers and reports covering aquatic vegetation surveys from 1960 to 2014 [31–36] (all data was published in Chinese). Aquatic vegetation surveys undertaken for this current study (from 1960 to 2014) covered (1) species number at the survey sites (east Taihu Lake) and (2) vegetative production (g/m^2^) of each type of vegetation (i.e., submerged macrophytes, floating-leaf macrophytes, and emerged macrophytes).

## 3. Results and Discussion

**Figure 2:**
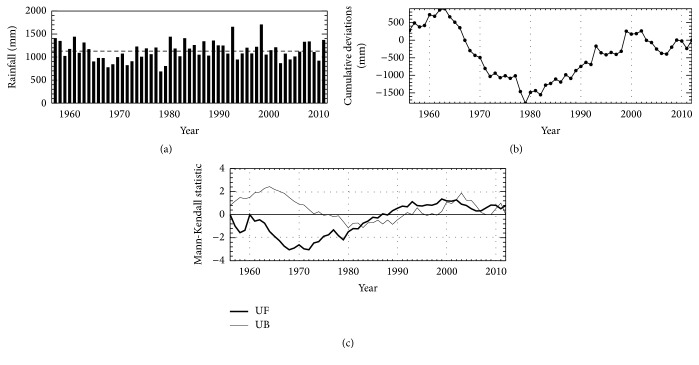
(a) Time series of total annual rainfall at Dongshan weather station (1956–2012). The horizontal line shows the average rainfall throughout the studied period. (b) Cumulative sum of deviations from the average rainfall. (c) Results of the Mann–Kendall test. The solid line is the UF statistic and the thin line is the retrograde series UB.

According to Figure 2(a), the maximum annual rainfall at studied station (1649.9 mm) occurred in 1999, while the minimum rainfall (680.1 mm) happened in 1978. The cumulative sums of deviations detected 3 periods (Figure 2(b)): 1956~1962/1963, 1963/1964~1978/1979, and 1979/1980~2012, which had average rainfalls of 1239.8, 959.7, and 1182.0 mm, respectively. Using percentiles (Table 1), the second period is classified as “dry” and the other two periods as “normal.” There is a slightly decreasing trend until approximately 1979, and a slight increase after that year (Figure 2(c)). Since 1980, the two statistics are around 0, which indicates that annual rainfall after 1980 fluctuated around the average value. In addition, the curve UF overlaps curve UB, which means that neither abrupt changes nor any trends were found in the annual rainfall series during the studied period (1956–2012).

**Figure 3:**
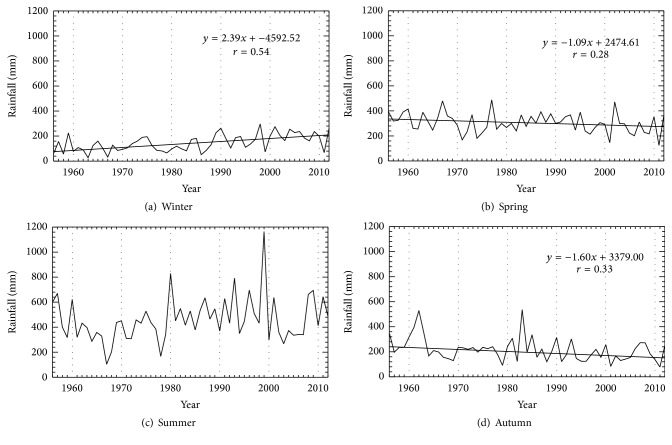
Time series of seasonal rainfall during the period 1956–2012. Linear trend lines are plotted for series with trends significant at 95% confidence level or greater.

The rainfall at the studied station shows an apparent seasonal variation (Figure 3). The peak rainy season is mainly summer, with the average depth 461.4 mm. The winter is relatively dry, with only about 145.9 mm in all three months. Table 1 summarizes the statistical results of annual and seasonal rainfall values. The main contribution to total annual rainfall comes from spring and summer values. Statistically significant decreases are found in spring and autumn in past several decades. On the other hand, the rainfall in winter exhibits statistically significant increase during 1956–2012. For summer, no significant trend was obtained. The maximum summer rainfall occurred in 1999, with a value 1163.8 mm, which is comparable to the average annual rainfall.

**Figure 4:**
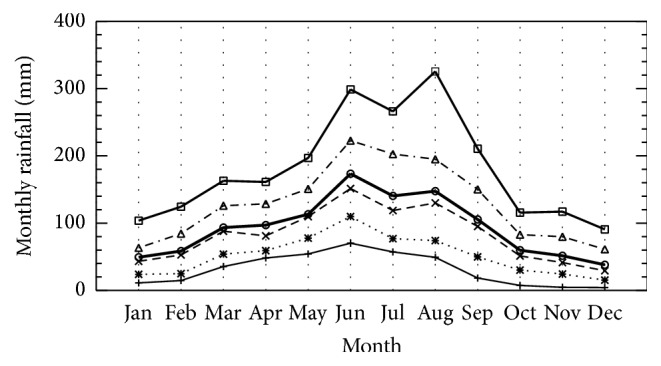
The percentiles of monthly rainfall are plotted as 10th (plus solid line), 25th (asterisk dot line), 50th (× dash line), 75th (triangle dash dot line), and 90th (square solid line). Average monthly rainfall is shown as circle thick solid line.

According to the monthly percentiles (Figure 4), monthly distribution of rainfall showed a maximum in June-August and minimum in December and January.  Table 2 provides the complete monthly statistical data for each month. In summer time, the 75th and 90th percentiles had the highest rainfall volumes, which implies that these three months have a greater probability of having the highest rainfall values. Interestingly, the maximum rainfall occurs mostly in June, except for 90th percentile. This indicates that the extreme rainfalls occurred more often in August. The “normal” values (between 25th and 75th percentiles) for June, July, and August were 109.8–222.7 mm, 76.9–202.7 mm, and 74.1–195.0 mm, respectively. In winter, the “normal” values were between 15.3 and 61.1 mm for December and between 23.9 and 63.3 mm for January. The median rainfalls are to be lower than the averages in all cases. The biases are larger in summer than those in other seasons.

**Figure 5:**
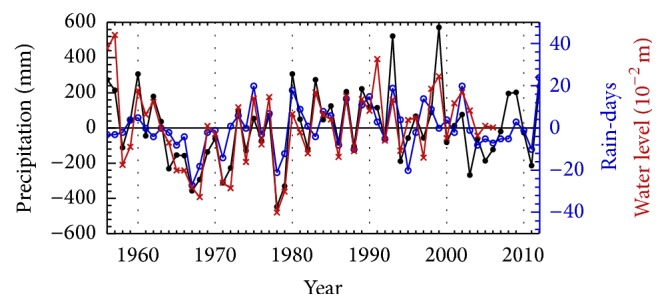
Relationship among the anomalies of annual rainfall (black solid circle), annual rain-days (blue open circle), and annual water level of Taihu Lake (red ×).

The annual average of rain-days at Dongshan station was 94. According to the relationship between the anomalies of annual rainfall and annual rain-days (Figure 5, black and blue curves), the number of rain-days was significantly positively correlated to total annual rainfall (Spearman *r* = 0.59, *n* = 57, *p* < 0.001). In dry period (1963/1964~1978/1979), the rain-days matched with the rainfall volume quite well. A decrement in rainfall depth usually corresponded with a decrease in number of rain-days. However, for those “normal” periods, annual rainfall and annual rain-days did not show this strong relationship. The rain-days remained low in spite of the high precipitation (such as, 1999, 2008, and 2009).

The variation of annual water level of Taihu Lake is significantly positively correlated with the annual rainfall (Figure 5, black and red curves). The correlation coefficient between them is 0.84 (*n* = 51, *p* < 0.001). It is reported that the changes of water level may limit the time for plastic responses of morphological and/or physiological traits in aquatic plants. In addition, if the water level change results in high level of disturbance, it may cause physical damage to the plants (e.g., stolon breakage, uprooting, or other damage, [37]), which eventually bring adverse impacts on plant growth [38, 39].

**Figure 6:**
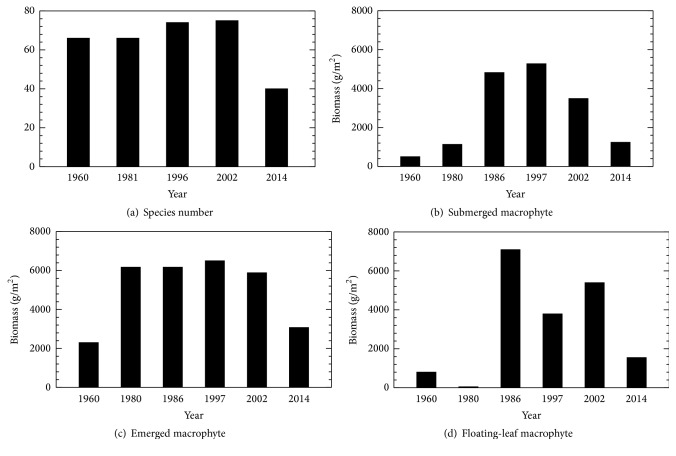
Species number (a), submerged macrophytes biomass (b), emerged macrophytes biomass (c), and floating-leaf macrophytes biomass (d) in east Taihu Lake from 1960 to 2014.

Since 1960, a total of 35 species of aquatic plants disappeared from Taihu Lake. The maximum total species number was observed in 1997 (75 species); however, only 40 species was observed in the most recent (2014) field survey (Figure 6(a)). The extinction species, such as* Potamogeton macckianus*, used to be widely distributed in the middle and lower Yangtze River. In the Taihu Lake, the most produced vegetation type is the emergent species, including* Phragmites australis* and* Zizania caduciflora*. Both species' biomass (g/m^2^) increased from 1960 to 1997; however, it decreased from 1997 to 2014 (Figure 6(c)). Similarly, the biomass of submerged macrophytes was also increased from 1960 to 1997 and decreased from 1997 to 2014 (Figure 6(b)). The species compositions were more complicated than emergent species. In 1980, the dominant submerged macrophytes were* Potamogeton malaianus*,* Vallisneria spiralis*, and* Hydrilla verticillata*; in 1996, the dominant submerged macrophytes were* Potamogeton macckianus*,* Elodea nuttallii* (exotic species),* Vallisneria spiralis*, and* Hydrilla verticillata*; in 2002, the dominant submerged macrophytes were* Elodea nuttallii*,* Ceratophyllum demersum*, and* Potamogeton macckianus*. For the floating-leaf species, there was a significant increase from 1960 to 2002 and a decrease from 2002 to 2014 (Figure 6(d)). The dominant species were* Nymphoides peltatum*,* Nymphoides indica*, and* Trapa bicornis*.

Previous studies [40, 41] revealed that eutrophication and its consequences of turbid water had led to the disappearance of aquatic vegetation in Taihu Lake. Similarly, our results show that since 2002, with the process of eutrophication, aquatic vegetation in east Taihu Lake significantly decreased in its biomass and species biodiversity. From a historical point of view, 1980 is the turning point of aquatic vegetation change in east Taihu Lake. From 1960 to 1980, the biomass of submerged macrophytes and floating-leaf macrophytes were relatively small compared with those in other periods. According to the investigation in long-term rainfall and water level measurements, sharp jumps were found in both 1960 and 1980, especially for the year 1980. In addition, based on Figure 2(b), the period 1960–1980 was classified as a “dry” period, in which all aquatic plants in Taihu Lake were dramatically reduced, especially for submerged macrophytes and floating-leaf macrophytes. These indicate that the variations of rainfall and water level may have an impact on the growth of the aquatic plant in Taihu Lake. The dry period may be not suitable for the growth of the aquatic plant. It is noteworthy that the comprehensive lake survey is relatively rare and more importantly most of these survey data were obtained in summer. Therefore, seasonal results about the aquatic vegetation in east Taihu Lake cannot be obtained from the current available data. In addition, it should be aware that there are necessary delays in the response of the aquatic vegetation growth to the water level changes. More detailed investigation in relationship between aquatic vegetation and the precipitation/water level is needed.

## 4. Conclusion

In this paper, the relationship between precipitation and aquatic vegetation succession in east Taihu Lake, China, is investigated by using long-term rainfall measurements (1956–2012), water level records (1956–2006), and also aquatic plant survey data from 1960 to 2014. The main findings are as follows:

1. Neither abrupt changes nor any trends were found in the annual rainfall series in Taihu Lake during the studied period (1956–2012). However, for seasonal variations, statistically significant decreases are found in spring and autumn, while the rainfall in winter exhibits statistically significant increase. No significant trend was obtained in summer. A “dry” period was detected in our studied period (1963/1964~1978/1979). The other years of the studied period are classified as “normal” period.
2. Total annual rainfall was significantly positively correlated to the number of rain-days (*r* = 0.59) and the water level (*r* = 0.84). However, the strong correlation between annual rainfall and rain-days only occurred in dry period. In those normal period, it is usually found that the rain-days remained low in spite of the high precipitation. Regarding the water level, the strong correlation exists throughout the studied period.
3. Our results indicate that the variations of rainfall and water level may have an impact on the aquatic plant in Taihu Lake. The dry period may be not suitable for the growth of the aquatic plant. All aquatic plants in Taihu Lake were dramatically reduced in the dry period, especially for submerged macrophytes and floating-leaf macrophytes.

## Figures and Tables

**Table 1:** Annual and seasonal statistical summary from monthly rainfall series (1956–2012) at Dongshan station.

Parameter	Annual	Winter	Spring	Summer	Autumn
Average (mm)	1127.7	145.9	303.8	461.4	216.7
Variance (mm^2^)	43171.9	4507.9	6210.3	31264.4	8768.7
SD (mm)	207.8	67.1	78.8	176.8	93.6
Minimum (mm)	680.1	27.1	124.3	103.4	78.4
10th percentile (mm)	860.1	65.3	214.0	297.4	122.7
25th percentile (mm)	999.6	90.0	246.7	341.4	154.6
50th percentile (mm)	1101.3	136.3	300.2	432.3	201.0
75th percentile (mm)	1252.7	196.6	364.7	549.0	249.8
90th percentile (mm)	1402.5	255.0	393.5	694.1	347.6
Maximum (mm)	1699.7	297.0	488.3	1163.8	536.6

**Table 2:** Statistical summary of monthly rainfall data at Dongshan (1956–2012).

Parameter	Jan	Feb	Mar	Apr	May	Jun	Jul	Aug	Sep	Oct	Nov	Dec
Average (mm)	49.3	58.5	93.5	97.1	113.2	173.4	140.4	147.5	105.5	59.7	51.5	38.1
Variance (mm^2^)	1361.6	1484.7	2366.1	2051.2	2392.2	11454.6	6472.8	8799.5	5926.2	2259.3	1460.7	875.5
SD (mm)	36.9	38.5	48.6	45.3	48.9	107.0	80.5	93.8	77.0	47.5	38.2	29.6
Minimum (mm)	0.0	0.0	14.9	30.4	24.5	23.6	19.1	0.1	6.5	0.0	1.3	0.0
10th percentile (mm)	11.2	14.6	35.5	48.2	54.2	70.2	57.2	49.2	18.4	7.4	4.6	4.3
25th percentile (mm)	23.9	24.9	53.9	59.1	77.8	109.8	76.9	74.1	49.8	30.2	24.4	15.3
50th percentile (mm)	43.3	52.8	88.0	81.1	109.7	151.4	118.8	130.0	95.1	51.2	41.4	29.3
75th percentile (mm)	63.3	84.5	125.9	128.7	150.9	222.7	202.7	195.0	150.2	82.9	79.8	61.1
90th percentile (mm)	103.7	124.4	162.9	161.2	196.9	298.7	266.1	325.4	210.6	115.6	117.1	90.7
Maximum (mm)	189.7	146.5	246.0	242.3	213.3	696.6	361.8	417.2	377.2	279.7	147.5	118.5
